# Comparison of treatment for idiopathic scoliosis based on 2D radiographic analysis and the GOSS system

**DOI:** 10.1186/1748-7161-8-S2-P9

**Published:** 2013-09-18

**Authors:** Jose Miguel Gomezl

**Affiliations:** 1 Gomez Orthotic Systems, St Petersburg, FL, USA

## Background information

Conservative treatment of the spine has been used for many more years than surgical treatment, especially for the management of idiopathic scoliosis. In general, the bio-mechanical plan of the orthotic treatment is based mainly on 2-dimensional radiographic studies from which the Cobb angle, Risser sign, pelvic obliquity and vertebral rotation are obtained. This is true despite the 3-dimensional nature of scoliotic deformities. “Gomez Orthotic Spine Systems” (GOSS) is a method based on the treatment of the patient as a whole structure. Taking into consideration the alignment in all three body planes, beginning with the localization of the lines of greatest stability: coronal center line (CCL), sagittal central line (SCL) and transverse center line of rotation (TCL). All these lines will emerge from the base of support on the feet, allowing the clinician to understand and quantify the overall alignment and the capacity of the patient’s balance and stability. The GOSS method consists of an established protocol that evaluates the patient using photometry, followed by the analysis of the ideal corrective shape in three dimensions of each patient. Using the protocol in the correct order, the evaluation/fabrication of a custom asymmetrical TLSO can be accomplished. 

## Purpose

The goal of this study was to compare and contrast the results of 2-dimensional radiographic and 3-dimensional photometry postural evaluation in a single case of idiopathic scoliosis before and after conservative treatment using the GOSS method. 

## Methods

This study was developed in the following phases:

(1) Pre-treatment evaluation: 3-dimension photometry postural and corrective shape evaluation based on the GOSS method vs. 2-dimensional radiographic evaluation. 

(2) Treatment: Fabrication of orthoses based on 3-dimensional corrective shape. 

(3) Post-treatment evaluation: 3-dimensional postural and corrective shape evaluation based on the GOSS method vs. 2-dimensional radiographic evaluation. 

(4) Analysis of pre/post evaluation results. 

### Pre-treatment evaluation

Sagittal plane: GOSS 1 centimeter anterior-decompensated; vs x-ray 10 centimeters posterior-decompensated

Transverse plane: GOSS 7.2 degrees clockwise at thoracic segment by Adam’s test vs. x-rays grade C under Nash-Moe method. 

Coronal plane: GOSS 3 centimeters to the right vs. x-ray no imbalance shown from C7-S1. 

## Results

Re-alignment to the most stable lines, SCL, TCL, and CCS, plus permanent reduction of the Cobb angle from 53 degrees to 28 degrees without an orthosis.

## Conclusions and discussion

The GOSS method of evaluation and treatment of idiopathic scoliosis is effective based on the results seen following treatment with an orthosis fabricated based on the 3-dimensional corrective shape of the patient presented in this case study. Under the right conditions with trained professionals, results like these would be ideal with a higher number of patients. 

